# Platelets modulate cardiac remodeling via the collagen receptor GPVI after acute myocardial infarction

**DOI:** 10.3389/fimmu.2023.1275788

**Published:** 2024-01-11

**Authors:** Friedrich Reusswig, Matthias Dille, E. Krüger, J. Ortscheid, Tobias Feige, S. Gorressen, J.-W. Fischer, Margitta Elvers

**Affiliations:** ^1^ Department of Vascular- and Endovascular Surgery, University Hospital Düsseldorf, Heinrich-Heine University, Düsseldorf, Germany; ^2^ Institute for Pharmacology and Clinical Pharmacology, Heinrich-Heine University, Düsseldorf, Germany

**Keywords:** platelets, glycoprotein VI, myocardial infarction, inflammation, remodeling, scar formation

## Abstract

**Introduction:**

Platelets play an important role in cardiovascular diseases. After acute myocardial infarction, platelets display enhanced activation and migrate into the infarct zone. Furthermore, platelets trigger acute inflammation and cardiac remodeling leading to alterations in scar formation and cardiac function as observed in thrombocytopenic mice. GPVI is the major collagen receptor in platelets and important for platelet activation and thrombus formation and stability. Antibody induced deletion of GPVI at the platelet surface or treatment of mice with recombinant GPVI-Fc results in reduced inflammation and decreased infarct size in a mouse model of AMI. However, the role of GPVI has not been fully clarified to date.

**Methods/Results:**

In this study, we found that GPVI is not involved in the inflammatory response in experimental AMI using GPVI deficient mice that were analyzed in a closed-chest model. However, reduced platelet activation in response to GPVI and PAR4 receptor stimulation resulted in reduced pro-coagulant activity leading to improved cardiac remodeling. In detail, GPVI deficiency in mice led to reduced TGF-β plasma levels and decreased expression of genes involved in cardiac remodeling such as Col1a1, Col3a1, periostin and Cthrc1 7 days post AMI. Consequently, collagen quality of the scar shifted to more tight and less fine collagen leading to improved scar formation and cardiac function in GPVI deficient mice at 21d post AMI.

**Conclusion:**

Taken together, this study identifies GPVI as a major regulator of platelet-induced cardiac remodeling and supports the potential relevance of GPVI as therapeutic target to reduce ischemia reperfusion injury and to improve cardiac healing.

## 1 Introduction

At sites of injury, platelets are essential mediators of hemostasis. However, they also play an important role in vessel occlusion leading to ischemic infarction under pathological conditions (1). At sites of atherosclerotic plaque rupture, uncontrolled platelet activation leads to acute myocardial infarction (AMI) because collagen is one of the procoagulant components of the atherosclerotic plaque and exposed to the flowing blood thus triggering platelet activation and the formation of a fibrin containing thrombus (2). After myocardial infarction, restoration of blood flow by thrombolysis and percutaneous coronary intervention (PCI) is important to re-canalize the obstructed vessel to limit cardiac damage but is accompanied by reperfusion injury (3). Thrombus fragmentation and distal embolization induced by thrombolysis and PCI might be triggered by platelets that might contribute to reperfusion injury by thrombus formation in reperfused vessels. Therefore, anti-platelet therapy is important in therapy and secondary prevention of AMI (4). In the last years, it became increasingly evident that platelets play an important role in the acute/inflammatory phase post AMI. The recruitment, transendothelial migration, and the activation of neutrophils and monocytes into the infarct border zone are promoted by secreted platelet cargo (e.g. serotonin, RANTES) and direct platelet interactions with inflammatory cells via proteins at the platelet surface such as P-selectin and the von Willebrand receptor GPIbα (1, 5–8). Furthermore, anti-platelet treatment of mice significantly reduced the recruitment of inflammatory cells into the infarcted myocardium (9). Enhanced platelet activation after AMI allows platelets to migrate into the infarct zone. Acute thrombocytopenia in mice leads to improved cardiac function along with reduced infarct size, both as the result of reduced inflammation. In addition, the interaction of platelets with fibroblasts modulates collagen composition in the left ventricle that was altered in platelet depleted mice leading to improved scar formation (10). This suggests that platelets not only trigger thrombus formation and inflammation but actively contribute to cardiac remodeling and scar formation after AMI. However, the molecular mechanisms and the responsible signaling pathways in platelets remain elusive. Recent publications point to an important role for the major collagen receptor of platelets, Glycoprotein (GP)VI, in platelet-mediated cellular responses post AMI. Antibody-induced loss of GPVI resulted in reduced infarct size 24h post AMI that was primarily due to improved microperfusion (11). Injection of a GPVI-Fc fusion protein into mice prevents platelet/endothelial interaction, reduced infarct size and the release of pro-inflammatory cytokines and preserved cardiac function in a mouse model of AMI (12, 13). However, the role of GPVI in AMI was not investigated in detail and the molecular mechanisms by which platelets affect cardiac remodeling remain elusive.

Here, we aimed to provide a detailed analysis of GPVI mediated cellular processes post AMI and the contribution in reperfusion injury. We believe that the identification of new therapeutic targets is important to limit cardiac damage induced by ischemia reperfusion injury and to prevent recurrent arterial thrombosis and ischemic events with reduced risk of bleeding complications as observed with the current anti-platelet therapy.

## 2 Materials and methods

### 2.1 Chemicals and antibodies

Heparin (Ratiopharm) was used as anticoagulant for blood collection. Platelets were activated with collagen-related peptide (CRP; Richard Farndale, University of Cambridge, Cambridge, UK), adenosine diphosphate (ADP; #A2754, Sigma-Aldrich), the thromboxane A2 analogue U46619 (U46; #1932, Tocris) or Par4 peptide (PAR4; St. Louis, Missouri, MO, USA). For flow cytometric analysis of platelets fluorophore conjugated antibodies labelling P-selectin (murine: #D200, Emfret Analytics; human: #555524, BD Biosciences), active integrin αIIbβ3 (murine: #D200; Emfret Analytics; human: #340507, BD Biosciences), GPIbα (#M040-2 Emfret Analytics), GPVI (#M011-1, Emfret Analytics), and integrin β3 (#M031-1, Emfret Analytics) were used. For PS exposure, AnnexinV (Cy™5, BD Pharmingen™, Cat. No. 559934) labelling of platelets was used.

### 2.2 Animals

Gene-targeted mice lacking GPVI (*Gp6^-/-^*) were provided by J. Ware (University of Arkansas for Medical Sciences) and backcrossed to C57BL/6 mice. For the generation of homozygous wildtype control (*Gp6^+/+^*) and *Gp6^−/−^* mice, heterozygous breeding partners were mated. Mice were maintained in an environmentally controlled room at 22 ± 1°C with a 12 h day-night cycle. Mice were housed in Macrolon cages type III with *ad libitum* access to food (standard chow diet) and water. All animal experiments were conducted according to the Declaration of Helsinki and approved by the Ethics Committee of the State Ministry of Agriculture, Nutrition and Forestry State of North Rhine-Westphalia, Germany (Reference number: AZ 81-02.04.2019.A270 & AZ 81-02.05.40.21.041).

### 2.3 Experimental model of acute myocardial infarction and reperfusion in mice

A closed-chest model of reperfused myocardial infarction was used in order to reduce surgical trauma and consequent inflammatory reaction from the intervention following ischemia and reperfusion (I/R) (10). Male mice in the age of 10 to 12 weeks underwent surgery. Mice were anesthetized with Ketamin (100 mg/kg body weight, Ketaset®, Zoetis) and Xylazin (10 mg/kg body weight, WDT) by a singular intraperitoneal (i.p.) injection before surgery. Euthanasia was performed by cervical dislocation.

After progressing successfully through anesthesia, the left anterior descending artery (LAD) was ligated for 60 min to induce MI 3 days post instrumentation. Coronary occlusion was achieved by gently pulling the applied suture tight until ST-elevation appeared on the ECG. Reperfusion was confirmed by resolution of ST-elevation. After 24 h of reperfusion, hearts were prepared and stained with TTC/Evans Blue–solution to stain the damaged area, separated in the area at risk (ischemic area) and the infarcted area. The ratios of the different areas were quantified digitally by video planimetry. To determine left ventricular function after MI, echocardiography was performed at different time points after I/R using Vevo 2100 ultrasound machine (VisualSonics Inc., Bothell, WA, USA) to measure different parameters, e.g., ejection fraction (%), cardiac output (mL/min), fractional shortening (%) and stroke volume (µL) with corresponding software.

### 2.4 Immunohistochemistry

At different time points after ischemia/reperfusion, hearts were flushed with cold heparin solution (20 U/mL, Roche, Basel, Switzerland), removed, paraffin embedded and cut into 5 µm sections.

For analysis of total cell migration into the infarcted heart tissue, paraffin-embedded heart sections were stained by Hematoxylin/Eosin (H/E) solution (Carl Roth, Karlsruhe, Germany) 24 h after myocardial infarction. The total number of cells migrated into the infarcted area was counted per visual field. Data are shown per *10^3^/mm^2^.

For analysis of scar formation, 21 days after ischemia/reperfusion paraffin-embedded heart sections were stained to determine collagen distribution. Scar formation was analyzed by staining with Gomori, Bouin’s (Sigma, Darmstadt, Germany) and hematoxylin solution (Carl Roth, Karlsruhe, Germany). Images were captured by Binocular Microscope (Nikon SMZ25, Tokyo, Japan), evaluated by Zen2 blue edition Software (Zeiss, Oberkochen, Germany) and the ratio of the infarct size to the total of the left ventricle was determined. To determine the amount of interstitial collagen, cardiac sections were stained by Picrosirius red staining (Morphisto, Frankfurt am Main, Germany) and Celestine blue solution (Sigma, Darmstadt, Germany) was used to stain the nuclei. Interstitial collagen was measured in percent by area fraction. Additionally, collagen density was analyzed by polarized light microscopy and evaluated by Image J software.

### 2.5 Immunofluorescence staining

To label myofibroblasts, αSMA-staining were conducted in sections of paraffin embedded hearts of *Gp6^+/+^* and *Gp6^-/-^* mice 7 days post AMI. After fixation, heart-sections were blocked by solution of 5% goat serum in PBS for 1 h and incubated with αSMA (Purified Rabbit anti-mouse αSMA, Abcam, Cambridge, UK, 1:100)-antibody or isotype control (Rat IgG2b kappa, eB149/10H5; Thermo Fisher Scientific Inc., Waltham, MA, USA, 1:100) o/n at 4°C. To label primary antibodies, incubation with Alexa Fluor 555 goat anti rabbit-IgG (Life technologies, Waltham, MA, USA, 1:100) for 1 h at RT followed. Nuclei were identified using DNA staining with 4,6 diamidino-2-phenylindole dihydrochloride (DAPI, Roche, 1:3000) for 5 min at RT. All fluorescence images were acquired using a confocal microscope AxioObserver (Carl Zeiss, Oberkochen, Germany) and supporting software.

### 2.6 Flow cytometry

Briefly, murine blood from retro-orbital plexus was collected in 300 μL heparin solution [20 U/mL in PBS] and total blood cell counts were analyzed by a hematology analyzer (Sysmex, Norderstedt, Germany). Heparinized murine whole blood was washed three times with Tyrode’s buffer by centrifugation at 650 g for 5 min. After washing the whole blood, samples were diluted in Tyrode’s buffer containing 1 mM CaCl_2_. For platelet activation analysis, samples were stimulated with indicated agonists and specifically labeled with antibodies against P-selectin and active integrin αIIbβ3 in a ratio of 1:10 for 15 min at 37°C. Reaction was stopped by adding 300 µL of PBS to all samples. To analyze glycoprotein (GP) expression, washed whole blood was labeled for GPIbα, GPVI, or integrin β3 in a ratio of 1:10 for 15 min at RT. For quantification, MFI (mean fluorescence intensity) values of the platelet specific FSC/SSC population was analyzed using a FACSCalibur flow cytometer (BD Biosciences). For detection of the PS exposure AnnexinV-staining was performed while binding buffer (10mM Hepes, 140 mM NaCl_2_, 2.5 mM CaCl_2_, pH 7.4) was used instead of PBS and CD42b was used as platelet specific marker.

At indicated time points after I/R, the formation of platelet-immune cell aggregates was determined via fluorescence based flow cytometry. Heparinized blood was washed twice with Tyrode’s buffer, centrifuged 5 min at 650 g, the supernatant was removed and only the cell-rich pellet was used for measurements. Samples were incubated with conjugated antibodies to label specifically platelets (GPIb- PE) and either neutrophiles (Ly6G-APC, Biolegend, San Diego, California, USA) or leucocytes (CD45-APC, BD Bioscience, Heidelberg, Germany). For analysis, the percentage of GPIb positive events in the neutrophil/leucocytes population was determined.

### 2.7 RT-PCR

For the analysis of endogenously expressed RNA levels, left ventricular heart tissue 6 h and 7 d after ischemia of *Gp6^+/+^* and *Gp6^-/-^* mice was used. Heart tissue was homogenized in 500μl cold TRIzol using a tissue homogenisator (Precellys® 24) and Precellys Lysing Kit (P000918-LYSK0-A) following manufacturer’s recommendations for soft tissue. Afterwards, RNA was isolated by Trizol/chloroform extraction and purification by RNAeasy Mini Kit (Qiagen, Hilden, Germany). Total RNA concentration was measured with an Eppendorf Bio Photometer® D30.purification by RNAeasy Mini Kit (Qiagen, Hilden, Germany) following the manufacturer´s protocol. Quantitative real-time PCR was performed using Fast Sybr Green Master Mix (Thermo Fischer Scientific) following the manufacturer’s protocol. The expression level of the target was normalized to glyceraldehyde-3-phosphate dehydrogenase (Gapdh) RNA expression levels as a control 6 h post I/R and hypoxanthine phosphoribosyltransferase 1 (Hprt1) 7 days post I/R. The following primer for RT-PCR were used **Table 1**:

**Table 1:** Primers used for detection of gene expression in RT-PCR.

Target gene	Primer sequence	
	forward (5′-3’)	reverse (5′-3’)
*Gapdh*	GGTGAAGGCGGTGTGAACG	CTCGCTCCTGGAAGATGGTG
*Hprt1*	AAGCTTGCTGGTGAAAAGGA	TTGCGCTCATCTTAGGCTTT
*Il1b*	AGCTTCCTTGTGCAAGTGTCTGAG	TGTTGATGTGCTGCTGCGAGAT
*Il6*	ACTCGGCAAACCTAGTGCGTTATG	ACATTCCAAGAAACCATCTGGCTAG
*Il10*	GCTATGCTGCCTGCTCTTACTGAC	CAGGGGAGAAATCGATGACAGCG
*Tnfa*	GCCCCCACTCTGACCCCTTT	GGGGCTGGCTCTGTGAGGAA
*Mcp1*	TTGTCACCAAGCTCAAGAGAGAGGT	SGGCATCACAGTCCGAGTCACACT
*Acta2*	ATGGAGTCAGCGGGCATC	CGTTCTGGAGGGGCAATGAT
*Col1α1*	AGGCGAAGGCAACAGTCG	TTTACACGAAGCAGGCAGGG
*Col3α1*	GCCTCCCAGAACATTACATACC	CTTGCTCCATTCC-CCAGTGT
*Postn*	TTCGTGGCAGCACCTTCAAA	GTCACCGTTTCGCCTTCTTT
*Cthrc1*	GCTGTCAGCGCTGGTATTTT	ACCCAGATGGCCACATCTAC
*Tgfb*	GAGCCAGAACGAGAAGTACCG	CCTCAAGACGAGCAATTTCATCA
*TgfbrI*	TCTGCATTGCACTTATGCTGA	AAAGGGCGATCTAGTGATGGA

### 2.8 Determination of plasma factors

For quantification of IL-1β, IL-6, and TGF-β in plasma after ischemia/reperfusion, blood was collected in plasma separator tubes and centrifuged 10 min at 10,000 g. The collected plasma was used for Enzyme-Linked Immunosorbent Assay (ELISA) to measure IL-6 and IL-1β 6 h post I/R (DuoSet Mouse IL-1β/IL-1F2/DuoSet Mouse IL-6/DuoSet Mouse). TGF-β in plasma of *Gp6^+/+^* and *Gp6^-/-^* mice was quantified 7 days after I/R (DuoSet Mouse TGF-β). All enzyme-linked immunosorbent assays were performed following the manufacturer’s protocol.

For quantitative analysis of cytokine levels 6 h post I/R, a murine cytokine panel analysis was performed with plasma collected as already described. To performed cytokine array (Proteome Profiler Mouse Cytokine Array Kit, Panel A; Catalog #: ARY006) instructions of the manufacture’s protocol were followed, while plasma samples were diluted 1:1.

### 2.9 Statistical analysis

GraphPad Prism 8 (version 8.4.3) was used to do the statistical analysis, and the data are provided as arithmetic means with SEM (Standard Error of Mean). The Sidak’s multiple comparison *post-hoc* test, a two-way or one-way ANOVA, or a unpaired student’s t-test were used to analyze statistical differences. Asterisks (*** = p<0.001, ** = p<0.01; * = p<0.05) are used to denote significant differences.

## 3 Results

### 3.1 Impaired platelet activation of GPVI deficient platelets after AMI

**Figure 1:**
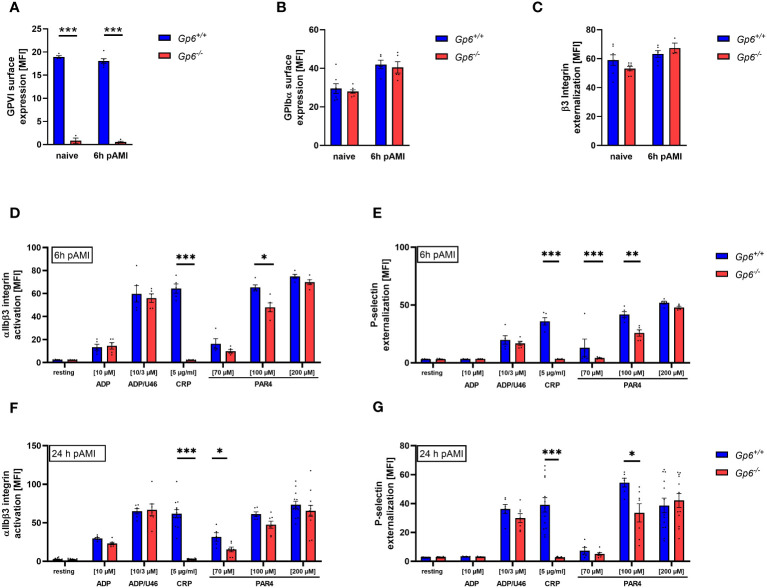
Ischemic/reperfusion (I/R) injury leads to impaired PAR4 signaling of GPVI deficient platelets. Flow cytometric analysis of blood platelets from naive *Gp6^+/+^* and *Gp6^-/-^* mice compared to different time points after I/R. Platelets were analyzed according to their specific FSC/SSC gate. **(A–C)** Analysis of glycoprotein surface expression of **(A)** GPVI, **(B)** GPIbα and **(C)** β3 integrin externalization in naive mice compared to 6 h post I/R (n = 3-8). **(D–G)** Platelets were analyzed with regard to αIIbβ3 integrin activation **(D, F)** and P-selectin externalization **(E, G)** upon stimulation with indicated agonists for 15 min. (n (6 h I/R) = 5; n (24 h I/R) = 5-12). Statistical significance was determined by unpaired student’s t-test. *p <.05, **p <.01, ***p <.001. Data shown as mean + SEM. CRP, collagen-related peptide; PAR4, PAR4 activating peptide; U46 (U46619), thromboxane A2 analogue.

Recently, others and we have shown that AMI in mice and in STEMI patients is accompanied by elevated activation and increased pro-coagulant activity of platelets (10, 14, 15). This allows platelet to migrate into the infarct zone and to modulate cellular processes post AMI such as inflammation and cardiac remodeling (10). To investigate, if GPVI is involved in these processes, we here analyzed GPVI deficient mice in experimental myocardial I/R. First, we determined blood cells counts, mean platelet volume (MPV) in GPVI deficient, control mice, and detect no alterations as shown in **Supplemental Tables 1** and **2**. Furthermore, we analyzed receptor exposure and platelet activation in naïve GPVI deficient mice and at different time points after AMI using flow cytometry (**Figure 1**). Glycoprotein (GP)VI, GPIbα and β3 integrin exposure at the platelet surface of GPVI deficient mice was not different when naïve mice were compared to mice 6h after AMI (**Figures 1A–C**). Furthermore, determination of GPVI exposure was almost absent in GPVI deficient mice confirming the deficiency of GPVI in these mice (**Figure 1A**). Next, we analyzed platelet activation by determination of active integrin αIIbβ3 and P-selectin exposure at the platelet surface (**Figures 1D–G**, **Supplementary Figure 1**). At 6h and 24h after I/R, we detected reduced platelet activation in response to CRP and to low/intermediate concentrations of PAR4 peptide stimulation using GPVI deficient platelets (**Figures 1D–G**). At later time points post AMI (5d and 21d), the defective response of GPVI deficient platelets following PAR4 peptide stimulation was no longer detectable (**Supplementary Figure 1**).

### 3.2 Reduced pro-coagulant activity of GPVI deficient platelets after AMI

**Figure 2:**
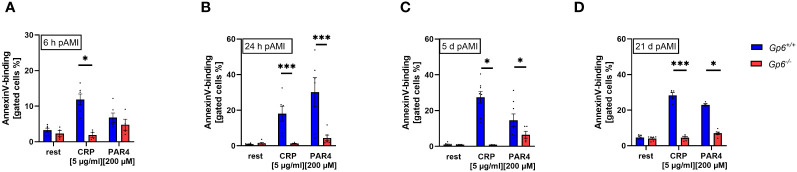
GPVI deficiency results in reduced PS exposure after I/R. PS exposure was determined via AnnexinV-binding of platelets in flow cytometric analysis. Whole blood platelets from naive, *Gp6^+/+^* and *Gp6^-/-^* mice were analyzed according to their specific FSC/SSC gate. At **(A)** 6h, **(B)** 24h, **(C)** 5d and **(D)** 21d post I/R AnnexinV positive platelets were analyzed after stimulation with indicated agonists (n = 4-8). Statistical significance was determined by two-way ANOVA with Sidak’s multiple comparisons test. *p <.05, ***p <.001. Data shown as mean + SEM. CRP, collagen-related peptide; Par4, Par4 activating peptide.

In line with reduced platelet activation, we detected reduced pro-coagulant activity as determined by Annexin-V binding to GPVI deficient platelets. At 6h after AMI, reduced pro-coagulant activity was only observed following CRP stimulation while PAR4 induced activation lead to unaltered AnnexinV-binding to GPVI deficient platelets (**Figure 2A**). In contrast, Annexin-V binding was significantly reduced after platelet activation with PAR4 peptide when platelets were isolated at 24h, 5d and 21d after I/R demonstrating strongly reduced pro-coagulant activity of GPVI deficient platelets post AMI (**Figures 2B–D**).

### 3.3 GPVI does not play a role in platelet-mediated inflammation after myocardial I/R injury in mice

**Figure 3:**
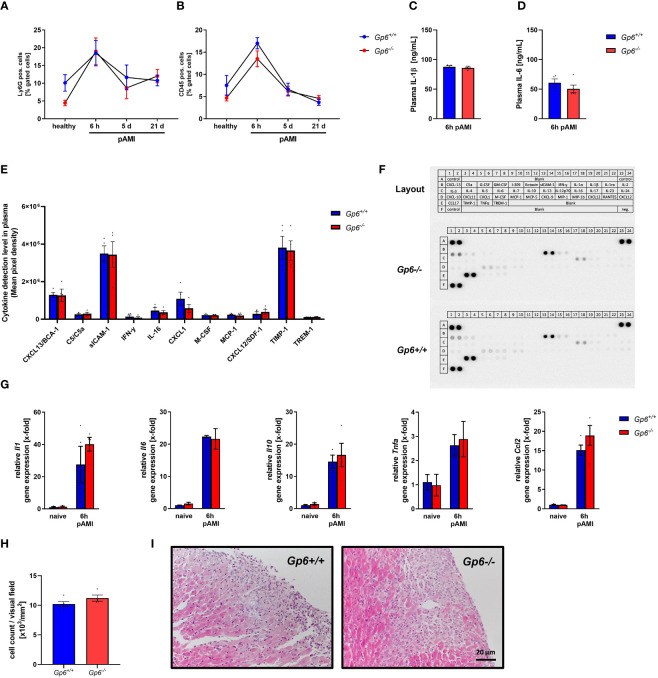
GPVI deletion has no impact on inflammatory responses after I/R injury in mice. **(A)** Flow cytometric analysis of platelet–neutrophil and **(B)** platelet–leukocyte aggregate formation in the blood of naïve mice compared to 6 h, 5 d and 21 d post I/R (n = 8-11). **(C)** Quantification of plasma interleukin (IL)-1β and **(D)** IL-6 concentration 6 h post I/R via ELISA (n= 5). **(E)** Plasma samples of *Gp6^+/+^* and *Gp6^-/-^* were analyzed via a murine cytokine panel 6 h post I/R to examine differences in pro-inflammatory response (n = 4). **(F)** Representative image of a cytokine panel. **(G)** Analysis of pro-inflammatory cytokine expression in heart tissue of naïve *Gp6^+/+^* and *Gp6^-/-^* mice and 6 h post I/R (n = 4). **(H)** Cardiac sections were stained with hematoxylin and eosin 24 h after I/R to analyze the migration of inflammatory cells into the infarct border zone with representative images shown in **(I)** (n = 4). Statistical significance was determined by two-way ANOVA with Sidak’s multiple comparisons test **(A, B, G)** or unpaired student’s T-test **(C–E, H)**. Data shown as mean + SEM.

To investigate the impact of platelet GPVI in the acute inflammatory response after ischemia and reperfusion, we determined the number of platelet-neutrophil (**Figure 3A**) and platelet-leukocyte conjugates (**Figure 3B**) in GPVI deficient and control mice at different time points. A strong increase in the formation of platelet conjugates with inflammatory cells was observed after 6h that declined at 5 and 21d post AMI (**Figures 3A, B**). However, no differences were detected between GPVI deficient and control mice. The determination of the acute phase cytokines IL-1β and IL-6 by ELISA also revealed no differences between groups (**Figures 3C, D**). Next, we used a cytokine array where we analyzed different cytokines and chemokines such as CXCL13, sICAM-1, CXCL-1, TIMP-1 and MCP-1 in the plasma of mice at 6h after AMI (**Figures 3E, F**). Again, no differences were observed between GPVI deficient and control mice. Quantification of gene expression of different acute phase cytokines and anti-inflammatory cytokine IL-10 using RNA isolated from the left ventricle of mice at 6h post AMI again revealed no differences between the groups (**Figure 3G**). The migration of inflammatory cells was comparable between GPVI deficient and control mice (**Figures 3H, I**), thus reflecting unaltered cytokine and chemokine levels in the left ventricle and in the plasma of mice as well as comparable numbers of platelet-leukocyte conjugates (**Figures 3A–G**).

### 3.4 Decreased TGF-β plasma levels and reduced stiffness-related gene expression leads to altered cardiac remodeling in GPVI deficient mice

**Figure 4:**
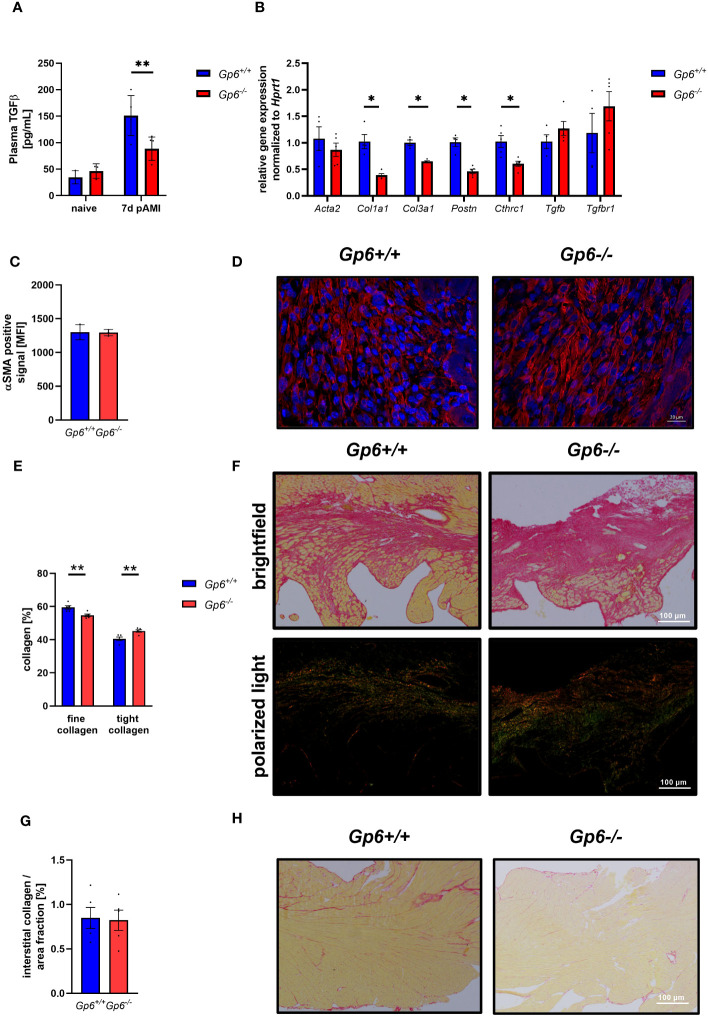
GPVI deficiency leads to altered cardiac remodeling 21d after I/R due to reduced TGFβ plasma levels. **(A)** Quantification of plasma TGFβ in naive *Gp6^+/+^* and *Gp6^-/-^* mice compared to 7 d post I/R (n (naive) = 3; n (7d) = 5-6). **(B)** Expression of genes related to cardiac remodeling and scar formation using heart tissue (left ventricle) from day 7 post I/R (n = 4-5). **(C)** Analysis of α-SMA content (red) in the infarct area and **(D)** representative images of the staining of *Gp6^+/+^* and *Gp6^-/-^* mice after 7d I/R. DAPI (blue) was used as cell nucleus marker. (n = 3). **(E)** Analysis of collagen composition and **(F)** representative images of Sirius-red staining of *Gp6^+/+^* and *Gp6^-/-^* mice after 21d I/R. In bright field images, yellow staining marks cytoplasm while red staining marks collagen. Polarized light was used to distinguish between thin (collagen type III in green) and dense (collagen type I in red-yellow) collagen fibers. (n = 4). **(G)** In the remote zone of transverse heart sections 21 days after I/R, interstitial collagen was analyzed in relation to fractional area. **(H)** Representative images of Sirius red staining in the remote zone after I/R are shown (n = 4). Statistical significance was determined by unpaired student’s T-test. **p <.01, *p <.001. Data shown as mean + SEM.

Incubation of cardiac fibroblasts with the supernatant of GPVI activated platelets leads to the up-regulation of activation markers such as periostin and gene expression of *Acta2*, *Col1a1* but not *Col3a1*. Consequently, the depletion of platelets after AMI resulted in altered cardiac remodeling and scar formation (10). To analyze if GPVI is a master regulator in these processes, we investigated cardiac remodeling in GPVI deficient mice (**Figure 4**). As shown in **Figure 4A**, TGF-β plasma levels were reduced 7d post I/R suggesting that GPVI induced platelet activation is responsible – at least in part – for the release of TGFβ (**Figure 4A**). The analysis of stiffness-related gene expression by RT-PCR revealed reduced expression of *Col1a1*, *Col3a1*, periostin and Collagen triple helix repeat containing 1(*Cthrc1*) but not *Acta2* or *Tgf-β* in heart tissue of the left ventricle at 7d post AMI (**Figure 4B**). After ischemia, resting fibroblasts become activated and transdifferentiate to myofibroblasts with enhanced expression of a-smooth muscle actin (αSMA) (16). To investigate if platelets not only trigger fibroblasts to secrete ECM proteins via GPVI but also modulate phenotypic switching of fibroblasts, we next investigated the number of αSMA positive cells in heart sections of mice. In line with unaltered gene expression of *Acta2*, we did not detect any differences in the number of αSMA positive cells in the left ventricle of mice after 7d of I/R (**Figures 4C, D**). However, altered TGF-β plasma levels and differences in gene expression resulted in altered collagen composition in GPVI deficient mice compared to controls with elevated levels of tight and reduced levels of fine collagen (**Figures 4E, F**). The quantification of interstitial collagen revealed no differences between the two groups (**Figures 4G, H**).

### 3.5 Reduced scar formation and improved left ventricular function after AMI as a consequence of altered cardiac remodeling in GPVI deficient mice

**Figure 5:**
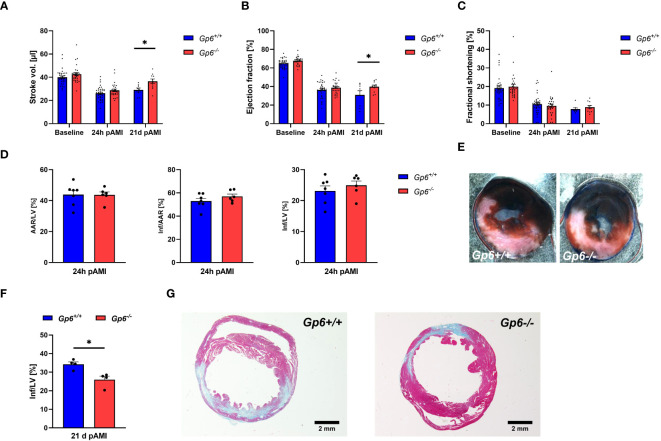
Genetic deletion of GPVI leads to reduced scar formation 21d post MI. *Gp6^+/+^* and *Gp6^-/-^* mice underwent I/R injury and were analyzed regarding heart function, infarct size and scar formation. **(A–C)** Echocardiographic analysis of **(A)** stroke volume, **(B)** ejection fraction and **(C)** fractional shortening of naive (baseline), *Gp6^+/+^* and *Gp6^-/-^* mice compared at 24 h and 21d after I/R. (n (baseline + 24h I/R) = 29-35; n (21d I/R) = 8-11). **(D)** Determination of infarct size and area at risk (AAR) as percentage of the left ventricle (LV), and infarct size (INF) as percentage of the area at risk analyzed by TTC staining 24 h after I/R with **(E)** representative TTC staining (blue: INF, red: AAR; white: naive tissue; n = 6-7). **(F)** Quantification of the infarct zone as percentage of the left ventricle in transversal cardiac sections stained with Gomori’s step one trichrome with **(G)** representative images 21 d after I/R (n=4). Statistical significance was determined by two-way ANOVA with Sidak’s multiple comparisons test **(A–C)** or unpaired student’s T-test **(D–F)**. *p <.05, Data shown as mean + SEM.

Next, we investigated the consequences of reduced activation and pro-coagulant activity of platelets and altered cardiac remodeling on infarct size, scar formation and left ventricular function. To this end, we determined stroke volume (**Figure 5A**), ejection fraction (**Figure 5B**) and fractional shortening (**Figure 5C**) at baseline, 24h and 21d post I/R. While no alterations between groups were observed at baseline and early time points (24h) after AMI, we detected improved heart function at 21d after AMI (**Figures 5A, C**). Furthermore, systolic volume was decreased in GPVI deficient mice at 21d post I/R while cardiac output, diastolic volume, heart rate and wall thickness were unaltered in these mice (**Supplemental Table 2**). Improved left ventricular function at late time points post ischemia/reperfusion was due to unaltered infarct size at early time points (24h, **Figures 5D, E**) but reduced scar formation at 21d post AMI (**Figures 5F, G**).

## 4 Discussion

In this study, we analyzed the consequences of genetically induced GPVI deficiency in mice on myocardial I/R injury to shed light on the impact of platelets on the inflammatory response and cardiac remodeling after AMI. As expected, we found abolished platelet activation (degranulation and αIIbβ3 integrin activation) in response to GPVI stimulation but also impaired activation following incubation with PAR4 peptide in the early phase but not at later time points (5 and 21d) after I/R. Reduced platelet activation did not alter acute inflammation. However, GPVI knockout mice showed altered cardiac remodeling leading to improved left ventricular function and reduced infarct size 21d post I/R.

Platelet activation plays an important role in myocardial I/R injury with platelet-derived P-selectin as a critical mediator in these processes (10, 15). Platelets have emerged as an important contributing factor in I/R injury by infiltrating into the myocardium and the formation of microthrombi, enhanced platelet-leukocyte conjugates and the release of potent vasoconstrictor and pro-inflammatory molecules (17). Thus, there is an essential use of antiplatelet drugs, such as aspirin and ADP receptor antagonists, to prevent acute coronary syndrome (ACS) (17, 18). The impact of GPVI on I/R injury post AMI has been studied by different approaches. Schönberger et al. injected the dimeric soluble fusion protein GPVI-Fc (Revacept) in mice. GPVI-Fc binds to collagen and protects against atherosclerosis (19) but also binds to activated endothelium mainly via vitronectin to prevent platelet-endothelial interactions after I/R (12). Treatment of mice with Revacept reduced infarct size 6h after reperfusion leading to improved left ventricular function at 28 days post AMI. In line with these results, Pachel and colleagues used mice where GPVI was deleted by antibody (JAQ1) treatment that leads to irreversible GPVI downregulation by JAQ1-induced ectodomain shedding (20). In JAQ1 treated mice, they detected reduced infarct size after 24h as a consequence of improved microperfusion (11). However, they did not investigate the consequences for cardiac function. Here, we detected no differences in infarct size at 24h but improved scar formation and reduced infarct size at 21d post AMI (**Figure 5**). This might be due to the here detected unaltered inflammation in GPVI deficient mice. However, Revacept as well as JAQ1 treatment of mice resulted in reduced inflammation: Treatment of mice with Revacept resulted in reduced gene expression of acute phase cytokines (IL-1, IL-6, TNF-α) in the tissue of the left ventricle 24h post AMI as detected by RT-PCR. The injection of JAQ1 reduced the infiltration of the myocardium with neutrophils but not with T-cells as observed after injection of JAQ1 into mice. However, the author did not quantify the number of migrated neutrophils in histological sections. These differences in the inflammatory response of mice after AMI might be due to the different models used in these studies. Treatment of mice with Revacept and JAQ1 was done in mice with 30 min ligation of the LAD that was followed by reperfusion using the open chest mouse model. In this study, we used the closed-chest model to avoid inflammatory responses induced by the surgery and ligated the LAD for 60 min to induce substantial cardiac damage. Thus, there are major differences in the experimental models used in the studies of Schönberger and Pachel and colleagues (11, 12) compared to the model used in this study. However, we have already shown that platelets are important in the inflammatory response after I/R as shown in mice with acute thrombocytopenia. These mice display reduced inflammation after AMI leading to reduced infarct size in these mice emphasizing the important role of acute inflammation for cardiac damage in the early phase after AMI (10). However, the data of this study provide evidence that platelet-induced inflammation in AMI is not mediated by GPVI.

Unfortunately, both studies that target GPVI in myocardial I/R did not analyze cardiac remodeling in detail. Schönberger et al. provided only infarct size at 28 days post AMI that was reduced by trend while Pachel and colleagues did not analyze JAQ1 treated mice at later time points after I/R (11, 12). This might be due to the fact that JAQ1 treatment of mice has to be done 6 days before I/R for normalization of platelet counts because JAQ1 induces transient thrombocytopenia in mice before platelets come back without GPVI at their surface (21). Overall, GPVI is not detectable for 2 weeks after JAQ1 treatment of mice (21). This is a clear limitation of the model because this short time window of normal counts of GPVI-depleted platelets does not allow the analysis of mice at later time points after I/R. Thus, cardiac remodeling cannot be addressed in JAQ1 treated mice after AMI.

Here, we detected strongly reduced pro-coagulant activity of GPVI deficient platelets (**Figure 2**) that together with significantly reduced TGF-β plasma levels (**Figure 4**) might be responsible for altered cardiac remodeling and scar formation. Different studies in the past have already shown that thrombin generation is associated with left ventricular impairment and worsened scar formation suggesting that thrombin generation represents an increased risk for patients with AMI (22–24). The here shown decreased pro-coagulant activity of GPVI deficient platelets in response to both, CRP and PAR4 peptide at early time points, might lead to reduced thrombin generation that probably accounts for improved scar formation and cardiac function in GPVI deficient mice. We already provided evidence that platelet activation by PAR4 peptide is involved in platelets activation after myocardial I/R (10). This together with results from Kolpakov and colleagues who provided evidence for a cardio-protective effect by PAR4 deficiency in experimental AMI point to an important role of the PAR4 signaling pathway in platelet mediated reperfusion injury after AMI (25). Interestingly, GPVI plays a role in PAR4 signaling because we observed reduced PAR4-induced platelet activation in GPVI deficient mice at early time points. It has already been shown that GPVI activation potentiated integrin activation induced by subthreshold concentration of thrombin in Syk deficient platelets suggesting that GPVI support thrombin-induced activation independent of Syk (26). Moreover, co-stimulation of platelets with thrombin and collagen leads to a highly enforced Ca^2+^ response suggesting that simultaneous G-protein coupled and GPVI receptor activation increased Ca^2+^ mobilization in platelets (27). In platelet-mediated clot retraction, PARs and GPVI cooperatively contribute to platelet-mediated clot contraction (28).

Beside the impact in AMI, deficiency of GPVI was found to be protective in thrombosis, pulmonary thromboembolism and thromboinflammation suggesting a dominant role of GPVI in arterial and venous thrombosis (29). Furthermore, GPVI plays a role in cancer-induced thrombosis because GPVI deficient mice show less metastatic foci after implantation of tumor cells into mice. this might be due to an interaction of GPVI with galectin from cancer cells or/and binding of GPVI to fibrin clots that are often found around tumor cells that express tissue factor (29).

To date, it becomes increasingly recognized that the extracellular collagen matrix plays an essential role in the healing and remodeling process after AMI (30). Here, collagen plays an important role and is synthesized by activated fibroblasts (31). Platelets are involved in these processes because they are able to activate primary cardiac fibroblasts that in turn respond with up-regulated gene expression of *Acta2* and *Col1a1* indicating a clear role of platelets in cardiac remodeling after I/R (10). TGF-β is probably the important mediator of platelet-induced fibroblast activation because reduced TGF-β plasma levels were detected in thrombocytopenic mice (10) and in GPVI deficient mice (**Figure 4A**). Yabanoglu and colleagues already detected elevated TGF-β levels in rat cardiac fibroblasts treated with platelet lysates leading to enhanced migration and proliferation of these cells (32). Interestingly, the supernatant of CRP-stimulated platelets induced elevated gene expression of *Col1a1* but not *Col3a1* (10). Here, we detected reduced gene expression of both, *Col1a1* and *Col3a1* in the left ventricle of GPVI deficient mice suggesting that the improved scar formation in platelet depleted (10) and in GPVI deficient mice (**Figures 4** and **5**) is due to these alterations in collagen content. However, enhanced collagen III has been described to be cardio-protective while collagen I levels are associated with left ventricular dysfunction suggesting that the collagen type I/III ratio is of importance for scar formation. This hypothesis was further supported by Xie and colleagues who claimed that an increase in the ratio of collagen type I/III leads to stiffening of the deposited collagens and enhances cross-linking capacity (33).

Interestingly, we recently showed that platelets modulate fibroblast phenotypic switching after AMI because they were able to induce gene expression of *Acta2* (αSMA) in primary cardiac fibroblasts (10). Although platelet induced gene expression was mediated by CRP activation that induced GPVI signaling of platelets, we did not detect any alterations in the number of αSMA positive cells in GPVI deficient mice (**Figure 4**). This suggests that either other GPVI-related mechanisms are involved in the transdifferentiation of fibroblasts to myofibroblasts or that there are differences in cellular processes in complex mouse models compared to isolated cardiac fibroblasts from naïve mice that were stimulated with the platelet supernatant *in vitro*. However, the number of mice analyzed in the study is very low; thus, the study represents a biological study to identify yet unknown roles of GPVI in cellular processes after AMI such as its role in cardiac remodeling. The beneficial effects of GPVI deficiency to limit I/R injury has to be verified in near future.

Taken together, this study supports the relevance of GPVI as a potential and even therapeutic target to reduce I/R injury and to improve cardiac remodelling. We here provide strong evidence that GPVI is much more than a platelet-activating signaling pathway important for platelet activation, thrombus formation and stability, and inflammation. Our results suggest that GPVI is a major regulator of platelet-induced cardiac remodelling due to GPVI mediated TGF-β release and elevated GPVI mediated pro-coagulant activity important for thrombin generation in AMI. Thus, interfering with GPVI activation improves cardiac remodeling by shifting fine to tight collagen within the scar leading to improved left ventricular function at later time points after I/R.

## Data availability statement

The original contributions presented in the study are included in the article/**Supplementary Material**. Further inquiries can be directed to the corresponding authors.

## Ethics statement

The animal studies were approved by Ethics Committee of the State Ministry of Agriculture, Nutrition and Forestry State of North Rhine-Westphalia, Germany. The studies were conducted in accordance with the local legislation and institutional requirements. Written informed consent was obtained from the owners for the participation of their animals in this study.

## Author contributions

ME: Conceptualization, Funding acquisition, Project administration, Resources, Supervision, Validation, Writing – original draft, Writing – review & editing. FR: Data curation, Formal Analysis, Investigation, Methodology, Validation, Writing – review & editing. MD: Formal Analysis, Investigation, Methodology, Writing – review & editing. TF: Investigation, Formal Analysis, Writing – review & editing. JO: Investigation, Writing – review & editing. SG: Investigation, Methodology, Writing – review & editing. J-WF: Supervision, Validation, Writing – review & editing. EK: Formal Analysis, Investigation, Methodology, Writing – review & editing.
