# Supplementary material for: Platelets modulate cardiac remodeling via the collagen receptor GPVI after acute myocardial infarction
Source: Front Immunol. 2024 Jan 11;14:1275788. doi: 10.3389/fimmu.2023.1275788 (PMC10808189; doi:10.3389/fimmu.2023.1275788)
Supplement: Supplementary file 1 [file DataSheet_1.pdf]

Supplementary Material

1 Supplementary Figures and Tables

1.1 Supplementary Figures

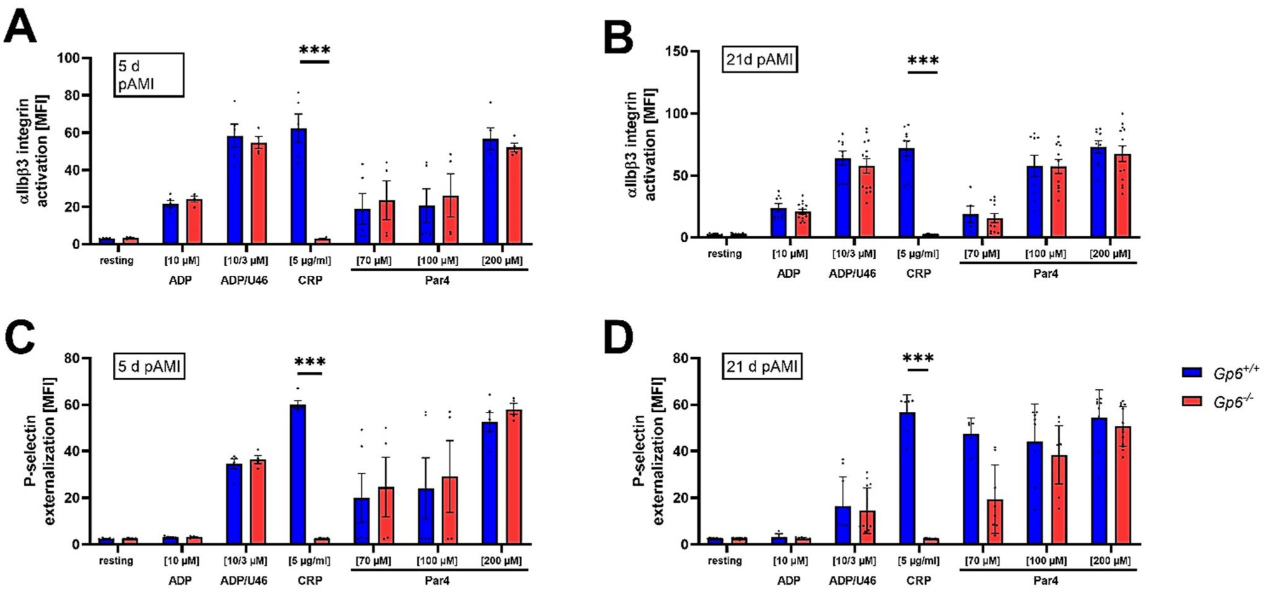

SUPPLEMENTAL FIGURE 1 | No differences in platelet activation following Par4 peptide stimulation at 5d and 21d post I/R. Flow cytometric analysis of blood platelets from *Gp6*$^{+/+}$ and *Gp6*$^{-/-}$ mice at 5d and 21d post AMI. Platelets were analyzed according to their specific FSC/SSC gate. (A, B) Platelets were analyzed with regard to αIIbβ3 integrin activation and (C,D) P-selectin externalization upon stimulation with indicated agonists for 15 min. (n (5 d I/R) = 4-5; n (21 d I/R) = 9-13). Statistical significance was determined by unpaired student's t-test. Data shown as mean + SEM. CRP = collagen-related peptide, Par4 = Par4 activating peptide, U46 (U46619) = thromboxane A2 analogue.

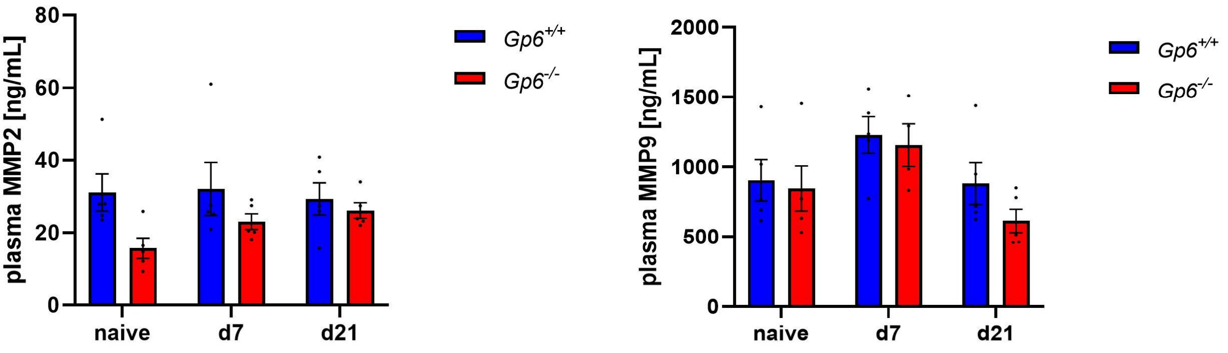

SUPPLEMENTAL FIGURE 2 | No differences in Matrix metalloproteinases (MMP)2 and 9 at 7d and 21d post I/R. (A) MMP2 and (B) MMP9 in the plasma of *Gp6*$^{+/+}$ and *Gp6*$^{-/-}$ mice was analyzed by ELISA, n=5. Statistical significance was determined by 2way ANOVA.

1.2 Supplementary Tables

SUPPLEMENTAL TABLE 1│Blood cell counts of *Gp6*$^{+/+}$ and *Gp6*$^{-/-}$ mice. Heparinized whole blood was analyzed via a hematology analyzer (Sysmex KN21). N (*Gp6*$^{+/+}$) = 8-10, n (*Gp6*$^{-/-}$) = 5-7. Two-Way ANOVA determined statistical significance with Sidak's multiple comparison test. Data are shown as mean + SEM. MPV = mean platelets volume, RBC = red blood cells, WBC = white blood cells. Time points: naïve or h post AMI.

Parameter	Time Point	*Gp6*$^{+/+}$	*Gp6*$^{-/-}$	*P*
platelets				
	naive	1.278 ± 0.02	1.375 ± 0.02	0.8566
	6 h	1.077 ± 0.01	1.106 ± 0.01	0.9998
	24 h	1.368 ± 0.04	1.552 ± 0,07	0.5815
	5 d	1.388 ± 0.02	1.478 ± 0.03	0.969
	21 d	1.32 ± 0.01	1.237 ± 0.02	0.93
MPV				
	naive	5.05 ± 0.01	5.117 ± 0.01	0.9464
	6 h	5.063 ± 0.01	5.05 ± 0.01	>0.9999
	24 h	5.111 ± 0.01	5,1 ± 0.03	>0.9999
	5 d	5.175 ± 0.02	5,1 ± 0.05	0.9653
	21 d	5.333 ± 0.02	5,431 ± 0.02	0.7453
RBC				
	naive	10.66 ± 0.2	10.95 ± 0.2	0.997
	6 h	9.452 ± 0.03	9.016 ± 0.09	0.9939
	24 h	13.96 ± 0.29	12.01 ± 0.86	0.277
	5 d	8.758 ± 0.07	9.154 ± 0.18	0.9969
	21 d	10.21 ± 0.11	9.858 ± 0.07	0.994
WBC				
	naive	7.812 ± 0.2	7.274 ± 0.19	0.978
	6 h	6.126 ± 0.26	6.102 ± 0.17	>0.9999
	24 h	4.06 ± 0.19	3.64 ± 1.24	0.9984
	5 d	4,73 ± 0.3	5,67 ± 0.48	0.9257
	21 d	6.689 ± 0.25	4.482 ± 0.13	0.0645

SUPPLEMENTAL TABLE 2│Echocardiographic analysis of heart function and wall thickness parameters of *Gp6*$^{+/+}$ and *Gp6*$^{-/-}$ mice post I/R. n (*Gp6*$^{+/+}$) = 34, n (*Gp6*$^{-/-}$) = 30 (baseline + 24h post AMI); n (*Gp6*$^{+/+}$) = 8, n (*Gp6*$^{-/-}$) = 11 (21d post AMI). Two-Way ANOVA determined statistical significance with Sidak's multiple comparison test. Data shown as mean + SEM. LVAWs = left ventricular anterior wall – systole; LVAWd = left ventricular anterior wall – diastole; LVPWs = left ventricular posterior wall – systole; LVPWd = left ventricular posterior wall – diastole. Time points: naïve or h post AMI.

Heart function

Parameter	Time Point	*Gp6*$^{+/+}$	*Gp6*$^{-/-}$	*P*
heart rate [BPM]				
	naive	509.4 ± 1.59	530.2 ± 1.69	0.3389
	24h	581.7 ± 1.86	591.1 ± 1.76	0.8682
	21d	534 ± 6.7	557.2 ± 3.49	0.7312
systolic volume [µL]				
	naive	21.82 ± 0.14	20.38 ± 0.15	0.9606
	24h	47.07 ± 0.36	45.49 ± 0.27	0.9491
	21d	78.29 ± 5.40	56.49 ± 1.41	0.0013
diastolic volume [µL]				
	naive	61.82 ± 0.28	63.84 ± 0.42	0.9527
	24h	73.29 ± 0.38	75.88 ± 0.44	0.9076
	21d	107.3 ± 4.97	102.0 ± 3.08	0.8747
cardiac output [mL/min]				
	naive	20.06 ± 0.09	22.53 ± 0.13	0.0588
	24h	14.36 ± 0.11	14.91 ± 0.15	0.9363
	21d	14.53 ± 0.56	14.93 ± 0.57	0.9955

Wall thickness

Parameter	Time Point	*Gp6*$^{+/+}$	*Gp6*$^{-/-}$	*P*
LVAWs [mm]				
	naive	0.843 ± 0.0026	0.8082 ± 0.0046	0.6716
	24h	0.6814 ± 0.0035	0.7434 ± 0.0049	0.2009
	21d	0.682 ± 0.0239	0.7717 ± 0.0186	0.4057
LVAWd [mm]				
	naive	0.6029 ± 0.0018	0.5742 ± 0.0034	0.5735
	24h	0.596 ± 0.0027	0.59 ± 0.0032	0.9928
	21d	0.6095 ± 0.0207	0.589 ± 0.0122	0.9581
LVPWs [mm]				
	naive	0.988 ± 0.0039	1.008 ± 0.0057	0.9325
	24h	0.8341 ± 0.0044	0.8727 ± 0.0045	0.6753
	21d	0.8296 ± 0.02	0.8811 ± 0.0162	0.8481
LVPWd [mm]				
	naive	0.6092 ± 0.0026	0.5766 ± 0.002	0.3604
	24h	0.603 ± 0.0024	0.6486 ± 0.0034	0.1122
	21d	0.629 ± 0.01	0.6179 ± 0.008	0.99
